# Resistance to cancer chemotherapy: failure in drug response from ADME to P-gp

**DOI:** 10.1186/s12935-015-0221-1

**Published:** 2015-07-15

**Authors:** Khalid O Alfarouk, Christian-Martin Stock, Sophie Taylor, Megan Walsh, Abdel Khalig Muddathir, Daniel Verduzco, Adil H H Bashir, Osama Y Mohammed, Gamal O Elhassan, Salvador Harguindey, Stephan J Reshkin, Muntaser E Ibrahim, Cyril Rauch

**Affiliations:** Institute of Endemic Diseases, University of Khartoum, Khartoum, Sudan; University of Münster, Münster, Germany; School of Veterinary Medicine and Science, University of Nottingham, Nottingham, UK; Faculty of Pharmacy, University of Khartoum, Khartoum, Sudan; H. Lee Moffitt Cancer Center, Tampa, FL USA; King Saud University, Riyadh, Kingdom of Saudi Arabia; Uneizah Pharmacy College, Qassim University, AL-Qassim, Kingdom of Saudi Arabia; Faculty of Pharmacy, Omdurman Islamic University, Khartoum, Sudan; Institute of Clinical Biology and Metabolism, Vitoria, Spain; Department of Biosciences, Biotechnologies and Biopharmaceutics, University of Bari, Bari, Italy

**Keywords:** Drug, Resistance, Pharmacokinetics, ADME, pH, MDR

## Abstract

Cancer chemotherapy resistance (MDR) is the innate and/or acquired ability of cancer cells to evade the effects of chemotherapeutics and is one of the most pressing major dilemmas in cancer therapy. Chemotherapy resistance can arise due to several host or tumor-related factors. However, most current research is focused on tumor-specific factors and specifically genes that handle expression of pumps that efflux accumulated drugs inside malignantly transformed types of cells. In this work, we suggest a wider and alternative perspective that sets the stage for a future platform in modifying drug resistance with respect to the treatment of cancer.

## Background

In US only, the newly diagnosed cancer patient is 1,665,540 every year and the estimated death is 585,720 [1] which are increasing as countries become more developed and more people reach advanced ages. Therefore, many efforts are being done in the war against cancer [2]. One of these efforts is the continuous development of new drugs and forms of chemotherapy.

**Figure 1:**
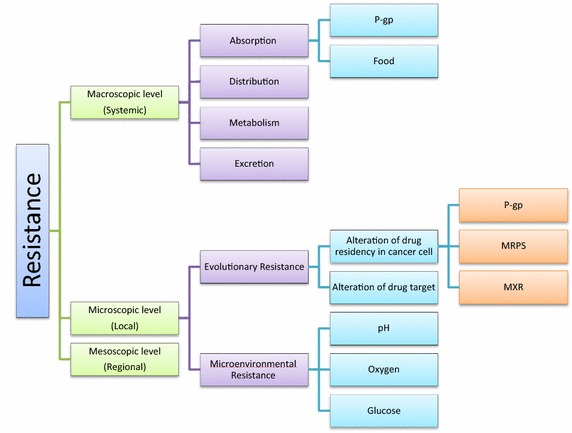
Diagrammatic representation describes that cancer chemotherapy resistance is a multi-factorial phenomenon that could be organized as multilevel structure.

In cancer, chemotherapy represents the backbone of treatment for many cancers at different stages of the disease. Therefore, chemotherapeutic resistance results in therapeutic failure and usually, (eventually) death. To address these limitations, many researchers focus on how cancer cells manipulate their genomes and metabolism to prevent drug influx and/or facilitate efflux of accumulated drugs, the so called: “*the neostrategy of cancer cells and tissues*” [3]. In this work, we show that drug resistance is a multifactorial phenomenon that requires attention to the host as well as the tumor and that such factors are organized at different levels (Figure 1).

## Macroscopic (systemic) resistance [host–related factors]

One of the major effects of host**-**related factors that determine the activity of the drug is pharmacokinetic. Pharmacokinetics is defined as the action of the body in response to drug and can be divided into the following consecutive steps: *A*bsorption, *D*istribution, *M*etabolism and *E*xcretion (ADME) [4]. Here we introduce the concept of “*Pharmacokinetic Resistance*” to describe the body-related factors that alter the effectiveness of the drug, so that it either does not reach its target and/or cannot accomplish its intended goal. Chemotherapeutics must be in contact with the tumor.

### Absorption

There are growing evidences suggesting that orally cancer chemotherapy is preferable to intravenous administration, because: (1) it is low-cost from the national health services perspective (i.e. does not require hospitalisation), (2) it can increase the drug’s antitumor activity by prolonging it’s time to clearance [5], (3) it reduces drug toxicity [6], (4) increase patients’ compliance and improve pharmacoeconomic issues [7–9]. However, to maintain a sufficient amount of orally administered chemotherapeutics, several factors should be taken into consideration:

#### P-gp

Permeability glycoprotein (P-gp), also known as multidrug drug resistance protein (MDR) is found along the gastrointestinal tract (GIT) [10], including the small intestine as primary site for the epithelial absorption of many orally administered drugs [11]. It has been shown that P-gp reduces the oral bioavailability of some anticancer drugs [12]. Concomitant administration of some of the antineoplastic agents leads to the overexpression of P-gp that results in bioavailability reduction of several these agents, e.g. Imatinib [13]. P-gp expression, in the gut, is a subject of interindividual variation due to either genetic polymorphism or pathologic condition [14, 15] and so fluctuates the bioavailability of several antineoplastic agents e.g. paclitaxel [12].

#### Food

The effects of food on drug absorption and bioavailability have been attracting attention very much and stimulated a long debate of whether fasting improved or worsened a drug’s bioavailability [16–18]. It has been shown that the half-life period of orally applied Topotecan is longer than that of intravenous administration. The administration of Topotecan with a high-fat breakfast shows a small decrease in the absorption rate but does not affect the extent of the absolute absorption [19]. St John’s wort, induces the expression of Pregnane X receptor, a xenobiotic or detoxification sensor, which reduces the efficacy of some antineoplastic agents, e.g. Irinotecan [4]. CYP3A4 is a metabolising enzyme (see below) which has been found in the intestine presumably as a defense strategy against xenobiotics [20]. It is well known that grapefruit juice abates the presence of CYP3A4, which is beneficial for the application of antineoplastic oral agents with low bioavailability [21]. Thus, it becomes apparent that the interaction between food and antineoplastic agents should be cautiously monitored to maintain a sufficient bioavailability [22–24].

### Distribution

**Figure 2:**
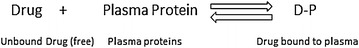
A drug is active while in unbound form. Therefore, the ability of ‎the drug to bind plasma proteins and tissue reduces its activity [221], ‎The drug’s activity is modulated by ifferences in the amount of plasma ‎proteins [222–224]. ‎

The “volume of distribution” (V_d_) of the drug is a hypothetical volume that outlines drug distribution into tissues [25, 26]. A higher V_d_ means that more of a drug penetrates into a tissue while it is more diluted (present at lower concentrations) in the plasma. The distribution of the drug between plasma and tissues relies on several factors. Some of them include:

1. *Gender* Metronidazole, a bactericide and protozoocide, also displays a potential activity as an anticancer drug, especially as a radiosensitizer in hypoxic regions [27], and shows a lower V_d_ in women [28] Furthermore, women are subject to pharmacokinetic variability during their menstrual cycle [29]. Therefore, it is critical to consider the gender factor also to age if the concentration of the drug in the plasma is to be determined or needs to be defined.
2. *Weight* Weight is a paramount factor that determines dose adjustment [30]. Cancer patients often lose weight during tumor progression [31–33]. Therefore, dosing based on body weight should be routinely re-adjusted to the current weight [34, 35].
3. *Plasma proteins* when a drug is available in the blood (plasma), the drug will be found either in free or bound form to plasma proteins (see Figure 2). Albumin and Alpha 1-acid glycoprotein are an example of drug-binding plasma proteins. While albumin carries acidic drugs, the alpha 1-acid glycoprotein will carry basic and neutral lipophilic drugs [36–38].

  Albumin: Albumin is an important binding protein in the blood. It is a powerful prognostic indicator reflecting diseases’ severity [39, 40], and its prognostic value is subject to gender differences [41]. It has been shown that etoposide is subject to individual variations (Population diversity) changes in the albumin serum concentration and/or age [42, 43].
  Alpha-1-acid glycoprotein (AGP or AAG) sometimes called orosomucoid (ORM) is an acute phase plasma alpha globulin glycoprotein. AGP is a critical determinant factor for the activity of several anticancer agents, e.g. imatinib [44]. Variation of AGP’s serum concentration also affects the anticancer activity of Gefitinib [45]. Wu et al. showed that the γ-secretase inhibitor RO4929097 (Notch signaling blocker) is bound in plasma with high affinity to AGP and can be competitively replaced by GDC-0449 (Hedgehog inhibitor). This consequently increases the availability of potentially active RO4929097 [46]. Therefore, it was suggested that AGP monitoring is critical to predict the pharmacodynamics response to a combined RO4929097/GDC-0449 treatment [46].
4. *Circadian rhythm* The circadian timing system comprises peripheral oscillators located in most tissues of the body and a central pacemaker located in the suprachiasmatic nucleus (SCN) of the hypothalamus [47]. The circadian rhythm has been implicated in the pathophysiology of several diseases [48–50], drug action [51, 52] and pharmacokinetics of drugs as well [53–55]. Plasma protein levels reach their minimum around 4:00 a.m. and start to increase around 8:00 a.m. [56]. This circadian rhythm can be masked at younger ages, but it aggravates and becomes clearer with aging [56]. Therefore, proper dosage timing should result in higher drug concentrations reaching the tumor site. It has been shown that alpha one acid glycoprotein (AGP) is subject to circadian rhythms [57]. In cancer, it has been demonstrated that Cisplatinum shows a considerable variability in its binding capacity day and night [58, 59]. Thus, circadian rhythmicity has a significant impact on a drug’s pharmacophore i.e. the active site of the drug molecule, and a delicate balancing between chronotolerability (minimum toxicity to host) and chronoefficacy (maximum cytotoxicity) is required [60]. Moreover, most of currently used anticancer agents act against highly proliferating cells, and since the basal metabolic rate is increased at night, it seems adequate to administer anticancer drugs at night instead of during the day.

### Metabolism

Drug metabolism does not reflect the conventional metabolic pathways, i.e. anabolic for biomass or catabolism for energy production. Instead, drug metabolism involves changing drug polarity and thus its hydrophilicity, to facilitate its excretion from the body. Drug metabolism occurs through two steps: the first involves reactions such as hydroxylation or oxidation [61, 62] of lipophilic drugs to make it vulnerable to the addition of glutathione, glucouronic acid or an amino acid [63, 64]. Although it is generally accepted that drug metabolism is a biological strategy of detoxification, some metabolism enzymes such as cytochrome P450 and glutathione S–transferase could be taken advantage of as they can activate certain anticancer drugs [65].

- A group of cytochrome P450 (CYP) enzymes responsible for the first step, the introduction of reactive or polar groups into xenobiotic groups [66]. CYP enzymes have been shown to activate some of the anticancer agents [67], as well as inactivate other anticancer drugs [68]. Overexpression of CYP450 in cancer patients might lead to resistance due to the rapid inactivation of the drug. Moreover, the presence of CYP450 shows interindividual variation [69–71] and so its detection, identification, and quantification prior to starting treatment is essential.
- Glutathione–S–Transferases (GSTs) are endogenous detoxifying enzymes [72] which mediate the second step of drug metabolism [73]. Overexpression of GST correlates with drug resistance [74–76]. This resistance could occur pharmacokinetically by metabolizing the drugs into inactive molecules [77]. Others suggest this resistance corresponds to detoxification via energy-dependent, transporter-mediated efflux of drugs or drug conjugates from the cell [78]. Also, GST generates resistance by suppressing apoptosis through its ROS-scavenging activity [79, 80] or via MAP kinase inhibition [81]. Conversely, GST is involved in the activation of certain drugs such as γ -Glutamyl- α -amino-(2- ethyl-N, N, N,N- tetrakis (2-chloroethyl) phosphoro-diamidate)-sulfonyl-propionyl)-(R)-(-) phenylglycine (TER286) [65], 6-mercaptopurine (6-MP) [82], and TLK-286 [83]. Because GST shows variability in its expression across populations [84–87], GST detection prior to chemotherapy could be utilized to inform established therapeutic strategies. Potential GST inhibitors include ethacrynic acid and buthionine sulfoximine [88]. These agents might be consumed concomitantly to keep GST in check in cancer and during cancer therapy. Interestingly, GST is subject to circadian rhythmicity that also affects the activity of 5–fluorouracil (5-FU) and Oxaliplatin [89–91].
- Extrahepatic metabolism: Typically, the liver plays a major role in drug metabolism. Drug-metabolizing enzymes are also present at other sites e.g. lung, gut, kidney, urinary bladder, skin [92–95]. Some of anticancer agent is a subjected into extrahepatic metabolism e.g. Oracin [95], and Paclitaxel could be subjected to extrahepatic metabolism too [96]. Extrahepatic metabolism also subjected to interindividual variation [95]. So, this issue should be addressed careful monitoring of these agents should discuss toward individualized chemotherapy [68, 97].

### Excretion

Excretion from the body is the final step in drug removal. Commonly, excretion of drugs occurs through two main routes: biliary and renal excretion.

- Biliary or bile duct excretion: MDR (ABC) mediates biliary excretion of xenobiotics [98]. Overexpression of ABC is correlated with an increase in biliary excretion [99–102]. Therefore, careful monitoring of ABC expression should be taken into account when defined anticancer drug is prescribed for the patients and this anticancer drug is knowingly excreted through the bile.
- Renal excretion: The kidney is the primary organ by which drugs are excreted. Interindividual renal drug excretion variability might be due to gender differences [103, 104] and ethnic differences [105]. So, changes in the glomerular filtration rate (GFR) have a direct effect on anticancer drug availability.

### Drug–drug interactions

Cancer chemotherapy is administered in the form of a cocktail, the combined application of several chemotherapeutics. Such a combination is designed to reduce toxicity and to decrease the likelihood of resistance. One drug alone would require a higher concentration and, so the side effects caused would be increased. Using a combination reduces the side-effects of each single drug as it can be applied at a significantly lower concentration [106]. A tumor consists of a heterogeneous population and it is commonly thought that using a “cocktail” of several agents will target different populations and thus reduce the selective pressure by using single agents (the use of only one single agent might kill one defined population and positively select a pre-adapted one that will remain and grow). Therefore, a combination therapy is useful in treating cancer. Conversely, it needs to be pointed out that co-administration of drugs might result in antagonism such that one drug may counteract or neutralize another one:

- Agents that target tumor vascularization: tumors require a blood supply for the provision of oxygen and nutrients [107], removal of metabolites [108] and to support metastasis [109]. It is widely assumed that administration of agents that target tumor vasculature (antiangiogenic therapy) eventually interrupts tumor progression. There are two classes of these agents (1) Angiogenesis inhibitors; they inhibit the tumor that has initiated the angiogenic process and (2) vascular disrupting agents that destruct the existing tumor vessels. Those agents might limit perfusion of cytotoxic drugs especially upon chronic administration [110]. Moreover, it is postulated that antiangiogenic therapy is useful in the management of resistance to chemotherapy [111], however, diminishing tumor vascularization may accelerate the adaptation to hypoxia while increasing the necrotic zone by accumulation of metabolites and so worsen tumor prognosis [112]
- NaHCO_3_ has been recently used systemically in the treatment of cancer [113]. It induces systemic alkalosis. By elevation of urine pH, methotrexate excretion is greatly enhanced. Therefore, NaHCO_3_ modulates the pharmacokinetics of methotrexate [114].
- Tamoxifen is a prodrug that needs to be metabolized to its active form by CYP2D6, CYP3A, CYP2B6 and CYP2C19 [115]. Some drugs, particularly from the group of selective serotonin reuptake inhibitors, inhibit CYP2D6 and so reduce the efficacy of Tamoxifen by decreasing amount of its active metabolites [116–118].
- Pravastatin, an HMG-CoA reductase inhibitor, is correlated with biliary excretion [119, 120] as a substrate of P-gp [121]. Also, it induces P-gp expression as well [122] and so may promote resistance to other compounds.

## Microscopic (local) resistance [tumor related factors]

The loss of ability of drugs to kill cancer cells could also be due to failure at the tumor site. Such disability could occur via several mechanisms. Some of them are:

### Evolutionary resistance

Also termed biochemical resistance [123, 124], acquired resistance [66, 125], active resistance [126], or extrinsic resistance [127]. Evolutionary resistance is an ancient type of resistance that can be found in bacteria even prior exposure to antibiotics [128–132]. Evolutionary resistance could occur either through interfering with drug resident time intracellularly and/or altering its site of action.

#### Alteration of drug residency in cancer cells

There are several proteins that alter drug residency in cancer cells. Some of these include:

#### P-gp

P-glycoprotein 1 (permeability glycoprotein, abbreviated as P-gp or Pgp) also known as multidrug resistance protein 1 (MDR1) or ATP-binding cassette sub-family B member 1 (ABCB1) or cluster of differentiation 243 (CD243). It is a glycoprotein that in humans is encoded by the ABCB1 gene [133, 134]. Commonly, P-gp is localized at the plasma membrane [135] of colon, jejunum, bile canaliculi, renal tubular cells, placenta, the luminal surface of capillary endothelial cells, testes, pancreas and blood–brain barriers (BBB) [135–138]. P-gp might have a role in the normal secretion of metabolites. P-gp also induces expression of CYP3A4 [139] that in turn may deactivate some anticancer drugs (see Table 1 that shows the pharmacological modulators of P-gp).

**Table 1:** Shows the pharmacological modulators of P-gp

Inducers	**Inhibitor**
Prazosin, Topotecan, Amprena [222], Rifampin, Phenobarbital, Clotrimazole, Reserpine, Isosafrole, Midazolam and Nifedipine [139, 223], Dexamethasone [224] Morphine [225], Retinoic acid [226], St John’s wort [227]	Carvedilol [228], Cyclosporine [229], Itraconazole [230], Ketoconazole [231], Synthetic opiates e.g. Meperidine, Methadone, Pentazocine [232], Tamoxifen [233, 234], Vandetanib [235], β-elemene [236]

In resistant cancer cell lines, P-gp is localized in the Golgi apparatus and the rough endoplasmic reticulum [140, 141]. Also, it is expressed in mitochondrial cristae [142, 143] to protect the accumulation of mitochondria [144] or prevent nuclear accumulation by expression of P-gp at the nuclear envelope [141, 145].

Expression of P-gp fluctuates with elevated expression level in untreated cancer into higher level upon relapse after chemotherapy and undetectable or low level in the expression in drug sensitive tumors [134, 146] which means there is no unifying theorem correlating expression of P-gp and cancer treatment.

##### MRPs

Multidrug resistance-associated protein MRP1 (ABCC1) was the first of the xenobiotic-transporting MRP-related proteins to be cloned and was identified based on its overexpression in a multidrug-resistant lung cancer cell line [147]. The MRP family consists of the four isoforms MRP1-4 [148, 149]. MRPs are similar to P-gp in that they are (I) capable of decreasing intracellular drug levels and (II) ATP-dependent [150]. Also, MRPs require glutathione GSH to extrude xenobiotics [151–154].

##### MXR

The Mitoxantrone resistance protein MXR or the Multixenobiotic resistance protein, also known as BCRP, ABCP and ABCG2, is one member of the ABC-superfamily that plays a role in trafficking biological molecules across cell membranes [155]. Expression of MXR might be an alternative strategy of resistance if cancer cells lack p-gp and MRP [156]. MXR preferentially extrudes large hydrophobic, positively charged molecules while others members of the MRP family can eject both hydrophobic uncharged molecules and water-soluble anionic compounds [157].

#### Alteration of drug target

When the drug reaches its target, another mechanism of resistance could be evolved somatically. Examples, which explain this mechanism of resistance, is:

- Methotrexate is a drug of choice for the treatment of rheumatoid arthritis [158–160]. Moreover, its activity against several types of tumors has been shown. It inhibits tumor cells via inhibition of the Dihydrofolate reductase enzyme (DHFR) which is a co-enzyme in DNA-methylation. Both, in vitro and in vivo studies show that the genomic amplification of the DHFR gene is reflected by extra copies of DHFR [161–163].
- 5-fluorouracil is a thymidylate synthetase inhibitor that is widely used in several types of tumors. Thymidylate synthetase is an enzyme used to generate thymidine monophosphate, which is subsequently phosphorylated to thymidine triphosphate for use in DNA synthesis and repair [164]. It has been postulated that one mechanism of resistance is the gain of extra copies of thymidylate synthetase genes [165, 166].

### Microenvironmental resistance

Cancer cells maintain a unique pH gradient; it is more acidic extracellularly and more alkaline intracellularly about normal tissues [167–169]. Such a pH gradient creates a unique environment around cancer cells. The tumor microenvironment becomes one of the cancer’s hallmarks [109]. Tumor microenvironment increases tumor’s fitness by blunting the immune system [167, 170], activating endogenous immunosuppressive strategies [171] and inhibiting the growth of the normal cell population. Moreover, the tumor microenvironment disables the activities of several chemotherapeutic agents resulting in resistance and failure in drug response [172] either through disturbing drug partitioning, sequestering it intracellularly [173, 174] or through induction of MDR expression [146].

There are several components of the tumor microenvironment that contribute to drug disability. Some of them include:

#### pH

Most anticancer agents are either weak bases or weak acids. Some of them are Zwitterions (see Table 2). Weakly basic anticancer drugs are ionizable at the interstitial fluid that decreases their partitioning, and if they cross the plasma membrane, they are sequestered into acidic vesicles (lysosomes). While weakly acid drugs increase their partitioning into the interstitial fluid, they will be rendered at cytosol due to intracellular alkalinity, and so they are slightly prevented from reaching their targets. This phenomenon is well known as “ion trapping mechanism” [123, 124, 175–177]. While basic drugs have reduced efficacy in an acidic microenvironment, Chlorambucil is a weakly acidic compound, and its cytotoxicity is enhanced by acidic microenvironments [178]. So, any attempt to induce intracellular acidification could be an avenue to both breaking through MDR and as an anticancer therapeutic approach it own [179].

**Table 2:** Shows pKa of some of commonly used anticancer agents

Drugs	pKa	Ionization behavior
Daunorubicin	8.3	Weak base
Doxorubicin	8.3	Weak base
Mitoxantrone	8.3	Weak base
Paclitaxel	Zwitterion	
5-Fluorouracil	7.76*	Weak acid
Cyclophosphamide	‎6.0‎	Weak acid
Chlorambucil	5.8	Weak acid
Cisplatin	5.06	Weak acid

* Although the pKa of 5-FU is higher but it considered as weak acid due to electrons withdrawn capacity due to Fluorine atom [124].

#### Oxygen

Intermittent hypoxia has been considered a suggested mechanism for the initiation of carcinogenesis [180–183] tumor evolution and progression [184–186] and metastasis [187].

Hypoxia somehow handles drug resistance via the following factors:

1. Most anticancer agents act to activate the apoptosis pathway, and the presence of free radicals are essential to promote this process [188]. Therefore, the absence of oxygen will diminish the activity of these drugs. Hypoxia does not only confer resistance to chemotherapy [189–192] but also to radiation [27].
2. Hypoxia induces genes expression that code for ABC-transporters [193] and so favors the developing of resistance of some of the anticancer drugs e.g. 5–fluorouracil [194].
3. Hypoxia also alters activity of some of metabolizing enzymes that are responsible for the activation and/or inactivation of some of the anticancer drugs e.g. conversion of paclitaxel metabolism into 6-α-hydroxypaclitaxel is reduced upon hypoxic conditions compared to normoxic conditions in HepaRG cells [195]. Therefore, hypoxia might alter therapeutic effectiveness.
4. Hypoxia is an important evolutionary determinant factor that shapes part of the tumor population to become hypoxia-adapted [108]. Hypoxia is associated with cellular senescence [196]. Chemotherapeutic agents which are designed to target cells that have high proliferation rates will fail even if they reach their site of action in sufficient amounts in poorly vascularized regions [197, 198]. Conversely, recent data suggests that hypoxia suppresses geroconversion (the conversion of arrested cells to senescence) [199] which make the role of hypoxia in tumorigenesis and tumor resistance still unclear.

#### Glucose

In the 1920s, Otto Warburg discovered that cancer have high aerobic glycolysis even in the presence of oxygen [200–202]. Recently, metabolic reprogramming of cancer has been adopted as one of the hallmarks of cancer [109]. Glucose, especially in high loads, induces over-expression of Sodium Hydrogen Exchanger 1 (NHE-1) resulting in the alkalinization of pHi [203] and in the induction of the metabolic transformation that aggravates the tumor microenvironment [27]. Moreover, glucose uptake is associated with tumor progression [204] as well as chemotherapeutic resistance [205]. Therefore, alteration of glucose transport [206], glucose deprivation, and fasting may enhance tumor sensitivity to chemotherapy [207]. However, targeting glucose also leads to interconversion of sensitive populations into more resistant populations, which may handle relapse [208]. So, targeting glucose as a potential strategy for overcoming resistance may be a dead end.

## Mesoscopic (physical, mechanical) or (regional) resistance [tumor—host interacting factors]

**Figure 3:**
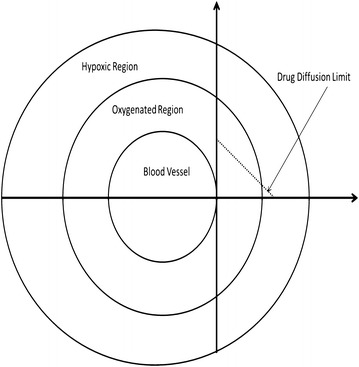
A hypothetical model describes the sphenoid tumor as mutli-‎habitat or multilayer shows that decrease of oxygen diffusion with drug gradients as a function of distance from the blood vessel.‎

The physics of the tumor site has a great impact on drug activity and results in drug resistance as follows:

1. Geometric, or vascular, resistance is a complex function of vascular morphology, i.e. the number of vessels of various types, their branching pattern, their diameter, and length [209]. It has been showing that upon clonal tumor expansion, tumor perfusion, and, therefore, the amount of drug to reaching its target is decreased [198, 210, 211]. Vascular modulating agents may alter vascular resistance and diminish the drugs reaching their targets [212] while by reducing the geometric resistance will enhance the activity of chemotherapeutic agents [213].
2. Blood viscosity is very significant especially for intratumoral blood flow [209]. Highly blood viscosity is an indicator for blood stasis and thus for the stagnation of drugs at certain sites. Moreover, inflammatory mediators at tumor sites might alter erythrocyte sedimentation rate and lead to greatly enhanced blood viscosity [214], at least at the tumor. Because blood flow has been correlated with oxygen diffusion kinetics [215], increasing blood viscosity may induces blood flow retardation and hypoxia becomes an adaptive strategy of survival especially for xeric phenotypes (cancer cells that grow distal from blood vessels) [108]. This also contributes to drug penetration into intratumor regions (see Figure 3). Anemia also might occur due reduction in hematocrit. In this regard, bone marrow suppression by chemotherapeutic agents should be taken into consideration.

### Co-resistance

The presence of the extracellular matrix (ECM) and a stroma are crucial for carcinogenesis. Both of this play a significant role in cancer progression, metabolism and the metastatic cascade [216]. In this context, the ECM plays a critical role in mediating drug resistance [217], either by acting as a physical barrier that impairs drug diffusion [218] and/or by cooperating with tumor cells to generate chemotherapy resistance [219, 220]. In this regard, this strategy of resistance could be called ECM-dependent resistance [220, 221].

## Conclusions

From bacteria to cancer, (multi) drug resistance is becoming a central issue and a significant challenge for medicine today. Although drug resistance is often studied at the single cell level, it is important to realize that the ability of a drug to interact with its target is more complex involving many body compartments. Also, over the past decade, there have been significant changes in our understanding of some fields from biology. Whereas, before, the key-lock model was supposed to uncover biology helping us to understand life, it is clear today that evolution theory needs to be introduced at the single cell level to clarify our understanding of some diseases including cancer. The famous key-lock model, as well as the long-awaited magic bullet to kill cancer, has to be revised accordingly. Therefore, we suggest reframing the concepts used in drug resistance in a more general context thereby dismantling the monolithic tone that the resistance is only a matter of genes. We propose that drug ineffectiveness results from tumor-host interactions and that a clear understanding of such an interaction open new opportunities not only for the discovery of new drugs but also for new therapeutic strategies to overcome the development and evolution of resistance to cancer chemotherapy.
